# Degenerate PCR Targeting the Major Capsid Protein Gene of HcRNAV and Related Viruses

**DOI:** 10.1264/jsme2.ME21075

**Published:** 2022-04-09

**Authors:** Michiko Takahashi, Kei Wada, Syun-ichi Urayama, Yuichi Masuda, Keizo Nagasaki

**Affiliations:** 1 Faculty of Science and Technology, Kochi University, 200 Otsu, Monobe-Otsu, Nankoku, Kochi 783–8502, Japan; 2 Department of Medical Sciences, University of Miyazaki, Kihara 5200, Kiyotake, Miyazaki, Miyazaki 889–1692, Japan; 3 Frontier Science Research Center, University of Miyazaki, Kihara 5200, Kiyotake, Miyazaki 889–1692, Japan; 4 Faculty of Life and Environmental Sciences, University of Tsukuba, 1–1–1 Tennodai, Tsukuba, Ibaraki 305–8577, Japan; 5 Graduate School of Integrated Arts and Sciences, Kochi University, 200 Otsu, Monobe, Nankoku, Kochi 783–8502, Japan

**Keywords:** eukaryotic algae, marine virus, phytoplankton, viral diversity, structure modeling

## Abstract

Heterocapsa circularisquama RNA virus (HcRNAV) is the only dinoflagellate-infecting RNA virus that has been isolated to date. We herein investigated the diversity of the major capsid protein gene of HcRNAV and related viruses using degenerate PCR and *in silico* ana­lyses. Diverse sequences related to HcRNAV were successfully amplified from marine sediments. Amplicons contained conserved and variable regions; the latter were predicted to be located on the outer surface of the capsid. Our approach provides insights into the diversity of viruses that are difficult to isolate in the environment and will enhance rapidly growing metagenome sequence repositories.

Viruses are more abundant than prokaryotes in aquatic environments (Wigington *et al.*, 2016) and are estimated to occur at an abundance of >10^6^ mL^–1^ (Bergh *et al.*, 1989; Suttle, 2005). They play important roles in structuring microbial communities, driving biogeochemical cycles, releasing predation pressure, and stimulating other trophic levels (Horas *et al.*, 2018; Kaneko *et al.*, 2021). Since the discovery of the first marine bacteriophage (from its host *Photobacterium phosphorium*) in 1955 (Spencer, 1955), a number of marine viruses that infect cyanobacteria and eukaryotic algae have been isolated and intensively studied (Safferman and Morris, 1963; Gibbs *et al.*, 1975; Coy *et al.*, 2018).

Nevertheless, only an extremely small proportion of the total diversity of marine viruses has been introduced into cultures and extensively examined. Dinoflagellates are a highly successful phytoplankton group comprising several hundred species (Li *et al.*, 2021), and a few dinoflagellate-infecting viruses have been isolated and investigated. Heterocapsa circularisquama RNA virus (HcRNAV) is the only dinoflagellate-infecting RNA virus that has been introduced into culture (Tomaru *et al.*, 2004). The whole genome of HcRNAV was sequenced in 2005 and two open reading frames, a replicase-polyprotein gene, and a major capsid protein (MCP) gene were identified. Since the amino acid sequence of the MCP gene has been shown to affect the intraspecific host range (Nagasaki *et al.*, 2005), its sequence data from other strains was a focus of interest. However, no sequences with close similarity to HcRNAV were present in the NCBI database at that time (Nagasaki *et al.*, 2005).

The growing number of metagenomic sequences in public repositories has enabled us to mine sequences similar to known HcRNAV isolates. A sequence homologous to a portion of the HcRNAV genome was initially reported in a putative single-stranded RNA virus originating from the metagenomic data of photosymbiotic corals; consensus sequences with the MCP gene of the HcRNAV strains were detected outside the previously reported hypervariable regions (Nagasaki *et al.*, 2005; Correa *et al.*, 2013). Several additional homologous sequences were subsequently obtained from metagenomic data (Shi *et al.*, 2016). Against this background, we investigated the diversity of HcRNAV and its related viruses in the environment by amplifying diverse MCP gene sequences using degenerate primers.

Degenerate primers containing all possible nucleotide sequences encoding a conserved amino acid motif have been used to reveal the diversity of a targeted family of viruses (Park *et al.*, 2011; Hopkins *et al.*, 2014; Li *et al.*, 2018; Tomaru and Kimura, 2020). However, the diversity of HcRNAV, the only isolated RNA virus that infects dinoflagellates, has not yet been elucidated through these experiments. The MCP gene of HcRNAV contains hypervariable regions (I to IV) associated with host specificity (Nagasaki *et al.*, 2005). Therefore, we attempted to obtain the MCP gene by designing primer sets for a PCR ana­lysis of conserved regions near the aforementioned hypervariable regions, in combination with high-throughput sequencing.

In the present study, eight sequences (three different MCP gene sequences from HcRNAV strains and five sequences from putatively related viruses obtained from metagenomic data) (Table S1) were used to design degenerate primers (Fig. S1) as previously described by Rose (2005). Marine sediments (depths of 0–15‍ ‍cm) were collected from Uranouchi Inlet, Kochi, Japan, and subdivided into five 3-cm layers (*i.e.*, 0–3, 3–6, 6–9, 9–12, and 12–15‍ ‍cm). Sediments were then subjected to total RNA extraction and reverse-transcribed. Amplicons obtained from degenerate PCR were sequenced on an Illumina MiSeq platform (300-bp paired-end reads). Sequencing reads were processed and amplicon sequence variants (ASVs) were generated and identified using Divisive Amplicon Denoising Algorithm 2 (Callahan *et al.*, 2016). Singleton ASVs were removed and the remaining ASVs were searched using BLASTn and BLASTp. ASVs with >85% homology to HcRNAV by the BLASTp search were labeled as HcRNAV-derived sequences and phylogenetically analyzed. The detailed method is described in the Supplemental material.

The results of the BLASTn search were as follows: 27 ASVs showed homology (>92%) to HcRNAV, 1 to another virus, and 11 to cellular organisms (bacteria, eukaryotes, and archaea), whereas 89 showed no significant search hits (Table S2). Nucleotide sequences were then translated to amino acid sequences, and multiple identical ASV sequences, due to synonymous substitutions, were observed (Table S3). Therefore, 128 nucleotide-based ASVs were condensed to 75 amino acid-based ASVs (ASVs coding for the same amino acid sequence were renamed as “ASV_XXXs”; see Table S3). The results of the BLASTp search using 75 amino acid-based ASVs were as follows: 13 ASVs showed homology to HcRNAV, 43 to other viruses, and 3 to cellular organisms, while 16 showed no significant search hits (Table S4). Beihai narna-like virus 9 and HcRNAV (Accession number: YP_009333317.1 and BCI98894.1, respectively) were the two most abundant ASVs with a relative abundance of 79.3 and 13.8%, respectively (Table S4). We then constructed a phylogenetic tree of ASVs with a read abundance ≥0.02%; ASVs were assigned to the HcRNAV (*Alvernaviridae*) and *Alvernaviridae*-like clades. However, the attribution of ASV_065 remains unknown (Fig. 1A). The Beihai narna-like and Beihai sobemo-like viruses used in the phylogenetic tree (Fig. 1A) were not derived from the *bona fide* narnavirus or sobemovirus, respectively. These viral sequences were identified from meta-transcriptome data and labeled “-like” based on their RNA-dependent RNA polymerase sequence (Shi *et al.*, 2016). We confirmed that the amplified sequences from the marine sediments were derived from either HcRNAV (*Alvernaviridae*) or HcRNAV-related (*Alvernaviridae*-like) viruses; however, we were unable to conclude whether the amplified sequences were derived from the narnavirus and/or sobemovirus (Fig. 1A).

Fig. 1B shows the read abundance of the ASVs obtained in each sediment layer based on the assignment information predicted in Fig. 1A. At a depth of 9–15‍ ‍cm, the abundance of *Alvernaviridae* (HcRNAV-derived reads) accounted for more than 50% of the total reads, whereas at a depth of 0–9‍ ‍cm, its abundance was minimal (<10%) and *Alvernaviridae*-like reads were dominant (Fig. 1B). Although read counts did not directly affect the quantitative composition of the amplified viruses in the environment due to amplicon sequencing biases, the dynamics of HcRNAV and related viruses may have been changed between depths of 6–9 and 9–12‍ ‍cm (Fig. 1B). We previously revealed the dynamics of HcRNAV in marine sediment over the last 100 years by combining radiometric dating and HcRNAV MCP gene amplicon sequencing (Takahashi *et al.*, 2021). However, based on radiometric dating, we needed to take into account that similarly collected sediment samples, such as those used in the present study, may have contained a mixed layer at a depth of 0–9‍ ‍cm. The application of the degenerate PCR designed in the present study to deeper sediments will provide insights into the diversity of ancient RNA viruses, which remain poorly understood.

We also focused on HcRNAV-derived reads (11 ASVs, Table S4) and performed a phylogenetic ana­lysis. HcRNAV clones were divided into UA- and CY-type clones based on host ranges (Tomaru *et al.*, 2004; Nagasaki *et al.*, 2005). Fig. 2A shows that the majority of HcRNAV-derived ASVs were assigned to UA-type clades, whereas only ASV_001s formed a monophyletic cluster with CY-type HcRNAV strains, and ASV_011s and ASV_088 were not assigned to known HcRNAV strain clades. Moreover, the amplicon contained both conserved (polymorphism of ≤1 residue) and variable (polymorphism of ≥2 residues) regions (Fig. 2B). Predictions of the amino acid sequence-based secondary structure indicated that conserved regions formed several β-strands. In contrast, the variable region was not predicted to form a secondary structure (Fig. 2B), implying that it may form flexible loop structures. Since the molecular structure of HcRNAV MCP is not currently available, we performed 3D-structure modeling using AlphaFold2 (Jumper *et al.*, 2021) (Fig. 2C). The putative trimeric model of the icosahedral capsid indicated that the flexible loop of the variable region was located on the outer surface of the virion. Due to high similarity in the amino acid sequence of the conserved region across the ASVs obtained, the conserved region may be involved in the formation of the basic capsid structure.

In conclusion, we herein successfully obtained diverse viral sequences from HcRNAV and related viruses and characterized the genetic diversity of HcRNAV. Although recent metagenomic technologies have rapidly uncovered viruses hidden in the environment (Shi *et al.*, 2016; Wolf *et al.*, 2020), amplicon-based approaches combined with degenerate primers and high-throughput sequencing may reveal the genetic diversity of target viruses at a higher resolution. The present results demonstrate the effectiveness of degenerate PCR for estimating the diversity of target viruses, which are difficult to isolate, in aquatic environments.

## Citation

Takahashi, M., Wada, K., Urayama, S., Masuda, Y., and Nagasaki, K. (2022) Degenerate PCR Targeting the Major Capsid Protein Gene of HcRNAV and Related Viruses. *Microbes Environ ***37**: ME21075.

https://doi.org/10.1264/jsme2.ME21075

## Supplementary Material

Supplementary Material

## Figures and Tables

**Fig. 1.**
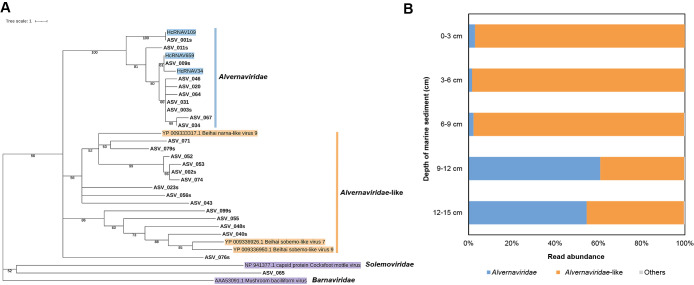
Overview of sequences obtained from the marine sediment. **A)** Phylogenetic tree based on the alignment of the amino acid sequences of amplicon sequence variants (ASVs) with ≥0.02% read abundance (Table S4). The tree was constructed using the neighbor-joining method. The scale bar indicates genetic distance. Numbers at the nodes are bootstrap values (1,000 replicates) >50%. **B)** Relative abundance of ASVs within each layer of the marine sediment based on assigned information in Fig. 1A. “Others” consists of ASV_065 whose attribution was unknown (see Fig. 1A) and ASVs with <0.02% read abundance.

**Fig. 2.**
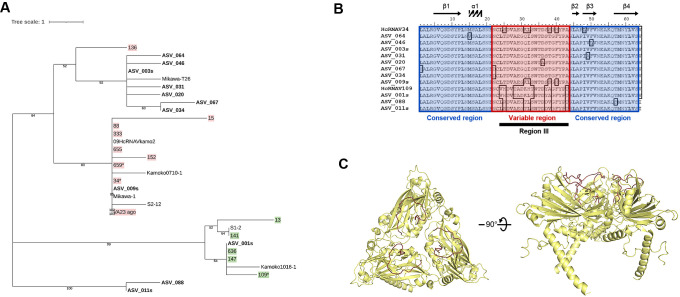
Characterization of HcRNAV-derived sequences obtained in the present study. **A)** Phylogenetic tree based on the alignment of the amino acid sequences of HcRNAV strains (Nagasaki *et al.*, 2005; Nakayama *et al.*, 2013; Nakayama and Hamaguchi, 2022), and HcRNAV-derived ASVs that showed >85% homology to HcRNAV in the BLASTp search (see Table S4) using the neighbor-joining method. The scale bar indicates genetic distance. Numbers at nodes are bootstrap values (1,000 replicates) >50%. Pink and green highlights represent the UA and CY types, respectively, which were identified using culture methods in previous studies (Nagasaki *et al.*, 2005; Nakayama *et al.*, 2013; Nakayama and Hamaguchi, 2022). The label with an asterisk represents reference strains for primer design. **B)** Alignment of HcRNAV-derived sequences amplified in the present study. Secondary structures were elucidated using PSIPRED (Buchan and Jones, 2019); β-strands are shown above the alignment as arrows. The blue and red highlighted regions indicate conserved regions (containing ≤1 polymorphism) and variable regions (containing ≥2 polymorphisms), respectively. “Region III” indicates the hypervariable region in the MCP gene identified by Nagasaki *et al.* (2005). **C**) The HcRNAV capsid 3D model was calculated using AlphaFold2 ver2.0 (Jumper *et al.*, 2021). Residues corresponding to the variable regions (Fig. 1B) are shown in red. Left, surface side view; right, side view (upper and lower sides represent outside and inside of the capsid, respectively).
