# Supplementary material for: Degenerate PCR Targeting the Major Capsid Protein Gene of HcRNAV and Related Viruses
Source: Microbes Environ. 2022 Apr 9;37(5):ME21075. doi: 10.1264/jsme2.ME21075 (PMC9763038; doi:10.1264/jsme2.ME21075)
Supplement: Supplementary file 1 — Supplementary Material [file 37_21075_s1.pdf]

Supplemental material

Degenerate PCR targeting the major capsid protein gene of HcRNAV and related viruses

Michiko Takahashi, Kei Wada, Syun-ichi Urayama, Yuichi Masuda, Keizo Nagasaki

Primer design

The degenerate primers were designed using j-CODEHOP in Base-by-Base (Tu et al., 2018) by aligning the MCP sequences of HcRNAV strains 34, 109, 659 and another five sequences that showed homology to HcRNAV (Table S1) with the codon usage previously reported (Hsiao et al., 2010). Next, a pair of degenerate primers (Fig. S1) were selected and synthesized (Fasmac Corporation, Kanagawa, Japan).

Sediment sampling and RNA extraction

A sediment core (top 54 cm depth) was manually sampled by a scuba diver at Menokuso Station (33.25.346N 133.23.522E) in the Uranouchi Inlet, Kochi, Japan on Aug. 22, 2019, and immediately stored at −80°C for 24 h. The frozen sediment was cut into 3-cm layers after freezing, as previously reported (Takahashi et al., 2021). Consequently, 2 g of sediment sample subdivided into 5 layers (0–3, 3–6, 6–9, 9–12, and

12–15 cm layers) was subjected to RNA extraction using the RNeasy PowerSoil Total RNA Kit (Qiagen, Hilden, Germany) according to the manufacturer's instructions.

Reverse transcription and degenerate PCR

The extracted RNA was subjected to reverse transcription using the Verso cDNA Synthesis Kit (Thermo Fisher Scientific, MA, USA). Briefly, 5 µL of sample RNA was mixed with 4 µL of 5× cDNA synthesis buffer, 2 µL of dNTP mixture (5 mM each), 1 µL of random hexamer (400 ng/µL), 1 µL of RT enhancer, 1 µL of Verso enzyme mix, and 6 µL of nuclease-free water. Reverse transcription was performed using the MiniAmp Plus System (Thermo Fisher Scientific, Waltham, MA) with the following reaction steps: 42°C for 30 min and denaturation at 95°C for 2 min. The resulting cDNA was used as a template for degenerate PCR using the ExTaq HS (Takara Bio Inc., Shiga, Japan). For the degenerate PCR, 4 µL of cDNA was mixed with 4 µL of 10× Ex Taq Buffer, 1.6 µL of dNTP mixture (2.5 mM each), 1 µM of degenerate primers, 0.4 µL of ExTaq HS, and 28.4 µL of nuclease-free water. Amplification was performed using the MiniAmp Plus, with 40 cycles of 94°C for 30 s, 60°C for 30 s and 60°C for 1 min. The PCR amplicons were electrophoresed at 100 V for 35 min on 1.5% (w/v) agarose gels in 1× TAE buffer.

Amplicon sequencing

Amplicons were purified using Ampure XP (Beckman Coulter Japan, Tokyo, Japan) according to the manufacturer’s instructions. Amplicon concentration was measured using Synergy H1 (BioTek Japan, Tokyo, Japan) and the QuantiFlour dsDNA System (Promega Japan, Tokyo, Japan), and subjected to library preparation. For the library preparation, 2 μL of amplicon was mixed with 10× Ex Taq buffer, 0.8 μL of dNTP mixture (2.5 mM each), 0.5 μL of primers (10 μM each; primers contained index sequences), 0.1 μL of Ex Taq HS, and 5.1 μL of distilled water. Amplification was performed with denaturation at 94°C for 2 min followed by 12 cycles of 94°C for 30 s, 60°C for 30 s, 72°C for 30 s, then elongation at 72°C for 5 min. The resultant library was amplified using Ampure XP, and the concentration was measured as described above. The library quality was checked using a fragment analyzer and dsDNA 915 Reagent Kit (Advanced Analytical Technologies, CA, USA) according to the manufacturer’s instruction. The libraries were sequenced on an Illumina MiSeq sequencer using the Reagent Kit v3 (300-bp, paired-end).

Sequencing data processing and phylogenetic analysis

For the data processing, those sequences where the initial nucleotides matched the primers were extracted using fastq_barcode_splitter in Fastx tool kit (Gordon & Hannon, 2010). After the primer sequences were deleted, sequence data was denoised and then amplicon sequence variants (ASVs) were generated using DADA2 pipeline (Callahan et al., 2016) in QIIME2 (Bolyen et al., 2019) with default parameters. The obtained ASVs, except singletons, were subjected to BLASTn/BLASTp search. Before the BLASTp search, the nucleotide sequences of each ASV were translated to amino acid sequence; if the amino acid sequence of any reads were identical, they were merged as "ASV_XXXs" (Table S3).

Phylogenetic analysis

The ASVs with a read abundance of ≥0.02% and the reference sequences HcRNAV34, HcRNAV109, HcRNAV659, Beihai narna-like virus 9, Beihai sobemo-like virus 7, and Beihai sobemo-like virus 9, (Acc. no. YP386496.1, BAE47072.1, BAU51723.1, YP009333317.1, YP009336926.1, and YP009336950.1, respectively) were aligned using ClustalW. Next, phylogenetic analysis was performed

using the neighbor-joining (NJ) method in MEGA X (Kumar et al., 2018). The phylogenetic tree was visualized using iTOL (Letunic & Bork, 2019).

The ASVs showing >85% of homology with the HcRNAV strains (yellow-colored row in Table S4) in the BLASTp search and the reference sequences of the HcRNAV strains from previous studies (Nagasaki et al., 2005; Nakayama et al., 2013; Nakayama & Hamaguchi, 2022) were aligned using ClustalW in MEGA X. A phylogenetic tree was constructed using the NJ method in MEGA X and visualized using iTOL (Letunic & Bork, 2019).

References

Bolyen, E., Rideout, J. R., Dillon, M. R., Bokulich, N. A., Abnet, C. C., Al-Ghalith, G. A., et al. (2019). Reproducible, interactive, scalable and extensible microbiome data science using QIIME 2. *Nature Biotechnology*, *37*, 852–857.

Callahan, B. J., McMurdie, P. J., Rosen, M. J., Han, A. W., Johnson, A. J. A., & Holmes, S. P. (2016). DADA2: High-resolution sample inference from Illumina amplicon data. *Nature Methods*, *13*, 581–583.

Gordon, A., & Hannon, G. J. (2010). FASTX-TOOLKIT, version 0.0.14. *Computer Program and Documentation Distributed by the Author*.

Hsiao, Y. Y., Lin, C. H., Liu, J. K., Wong, T. Y., & Kuo, J. (2010). Analysis of Codon Usage Patterns in Toxic Dinoflagellate Alexandrium tamarense through Expressed Sequence Tag Data. *Comparative and Functional Genomics*, *2010*, 138538.

Kumar, S., Stecher, G., Li, M., Knyaz, C., & Tamura, K. (2018). MEGA X: Molecular Evolutionary Genetics Analysis across Computing Platforms. *Molecular Biology and Evolution*, *35*, 1547–1549.

Letunic, I., & Bork, P. (2019). Interactive Tree Of Life (iTOL) v4: recent updates and new developments. *Nucleic Acids Research*, *47*, W256–W259.

Nagasaki, K., Shirai, Y., Takao, Y., Mizumoto, H., Nishida, K., & Tomaru, Y. (2005). Comparison of genome sequences of single-stranded RNA viruses infecting the bivalve-killing dinoflagellate Heterocapsa circularisquama. *Applied and Environmental Microbiology*, *71*, 8888–8894.

Nakayama, N., Fujimoto, A., Kawami, H., Tomaru, Y., Hata, N., & Nagasaki, K. (2013). High interaction variability of the bivalve-killing dinoflagellate Heterocapsa circularisquama strains and their single-stranded RNA virus HcRNAV isolates. *Microbes and Environments*, *28*, 112–119.

Nakayama, N., & Hamaguchi, M. (2022). The importance of the genetic diversity of the HcRNAV ssRNA virus in the viral-based bloom control of the dinoflagellate Heterocapsa circularisquama. *Aquaculture* , *546*, 737318.

Takahashi, M., Wada, K., Takano, Y., Matsuno, K., Masuda, Y., Arai, K., … Nagasaki, K. (2021). Chronological distribution of dinoflagellate-infecting RNA virus in marine sediment core. *The Science of the Total Environment*, *770*, 145220.

Tu, S. L., Staheli, J. P., McClay, C., McLeod, K., Rose, T. M., & Upton, C. (2018). Base-by-base version 3: New comparative tools for large virus genomes. *Viruses*, *10*, 637.

Table S1 Reference sequences for primer design.

Subgroup	Virus name	Target region	Accession number
Dinornavirus	Heterocapsa circularisquama strain 34	major capsid protein	YP_386496.1
Dinornavirus	Heterocapsa circularisquama strain 659	major capsid protein	BAU51723.1
Dinornavirus	Heterocapsa circularisquama strain 109	major capsid protein	BAE47072.1
unclassified Riboviria	Beihai narna-like virus 9	hypothetical protein	YP_009333317.1
unclassified Riboviria	Beihai sobemo-like virus 7	hypothetical protein 2	YP_009336926.1
unclassified Riboviria	Beihai sobemo-like virus 8	putative capsid protein	YP_009336986.1
unclassified Riboviria	Beihai sobemo-like virus 9	hypothetical protein 2	YP_009336950.1
unclassified Riboviria	Beihai weivirus-like virus 7	hypothetical protein	YP_009337048.1

Table S2 Read frequency at each sample subdivision depth and BLASTn hit table of amplicons obtained from marine sediment.

ASV ID	Read count in each depth of sediment					Subtotal reads	Top hit of BLASTn search			
	0-3 cm	3-6 cm	6-9 cm	9-12 cm	12-15 cm		Target	Identity (%)	E-value	Accession number
ASV_001	178	176	179	2618	1095	4246	Heterocapsa circularisquama RNA virus, isolate: HcRNAV109	97.409	1.57E-86	AB218609.1
ASV_002	19673	23111	18015	5038	3517	69354	Nohit			
ASV_003	887	483	626	13037	7850	22883	Heterocapsa circularisquama RNA virus, isolate: HcRNAV659, capsid protein	95.337	7.37E-80	LC120626.1
ASV_004	10419	8351	10842	2205	3437	35254	Nohit			
ASV_005	75	0	0	1386	746	2207	Heterocapsa circularisquama RNA virus, isolate: HcRNAV109	96.891	7.32E-85	AB218609.1
ASV_006	6930	7316	5815	4547	2129	26737	Nohit			
ASV_007	0	0	0	181	262	443	Heterocapsa circularisquama RNA virus, isolate: HcRNAV109	96.891	7.32E-85	AB218609.1
ASV_008	0	0	0	199	0	199	Heterocapsa circularisquama RNA virus, isolate: HcRNAV109	96.891	7.32E-85	AB218609.1
ASV_009	32	20	38	276	0	366	Heterocapsa circularisquama RNA virus, isolate: HcRNAV659, capsid protein	100	7.21E-95	LC120626.1
ASV_010	529	795	0	0	0	1324	Nohit			
ASV_011	48	21	0	964	136	1169	Heterocapsa circularisquama RNA virus, isolate: HcRNAV109	92.632	7.47E-70	AB218609.1
ASV_012	0	1018	0	0	0	1018	Nohit			
ASV_013	326	328	199	0	0	853	Nohit			
ASV_015	0	0	0	276	307	583	Nohit			
ASV_016	0	0	0	452	0	452	Heterocapsa circularisquama RNA virus, isolate: HcRNAV659, capsid protein	94.819	3.43E-78	LC120626.1
ASV_017	0	0	416	0	0	416	Nohit			
ASV_018	397	0	0	0	0	397	Nohit			
ASV_019	172	210	0	0	0	382	Nohit			
ASV_020	0	0	0	373	0	373	Heterocapsa circularisquama RNA virus, isolate: HcRNAV659, capsid protein	94.819	3.43E-78	LC120626.1
ASV_021	162	43	32	87	0	324	Nohit			
ASV_022	143	181	0	0	0	324	Nohit			
ASV_023	26	0	262	21	0	309	Nohit			
ASV_024	0	0	0	0	296	296	Heterocapsa circularisquama RNA virus, isolate: HcRNAV659, capsid protein	94.819	3.43E-78	LC120626.1
ASV_025	288	0	0	0	0	288	Nohit			
ASV_026	0	0	23	0	0	23	Nohit			
ASV_027	143	0	129	0	0	272	Nohit			
ASV_030	0	0	232	0	0	232	Nohit			
ASV_031	0	0	0	0	228	228	Heterocapsa circularisquama RNA virus, isolate: HcRNAV659, capsid protein	94.819	3.43E-78	LC120626.1
ASV_032	0	0	0	12	0	12	Nohit			
ASV_033	174	0	0	0	0	174	Nohit			
ASV_034	0	0	0	0	174	174	Heterocapsa circularisquama RNA virus, isolate: HcRNAV659, capsid protein	94.819	3.43E-78	LC120626.1
ASV_035	0	0	0	0	170	170	Heterocapsa circularisquama RNA virus, isolate: HcRNAV659, capsid protein	99.482	3.36E-93	LC120626.1
ASV_036	0	0	0	0	149	149	Heterocapsa circularisquama RNA virus, isolate: HcRNAV659, capsid protein	95.876	1.58E-81	LC120626.1
ASV_037	0	0	0	135	0	135	Nohit			
ASV_038	0	0	0	135	0	135	Heterocapsa circularisquama RNA virus, isolate: HcRNAV659, capsid protein	94.819	3.43E-78	LC120626.1
ASV_039	0	0	0	0	129	129	Heterocapsa circularisquama RNA virus, isolate: HcRNAV109	97.409	1.57E-86	AB218609.1
ASV_040	0	15	112	0	0	127	Nohit			
ASV_041	0	0	0	115	0	115	Nohit			
ASV_042	0	0	0	0	104	104	Heterocapsa circularisquama RNA virus, isolate: HcRNAV659, capsid protein	94.819	3.43E-78	LC120626.1
ASV_043	4	4	94	0	0	102	Nohit			
ASV_045	0	7	0	0	0	7	Nohit			
ASV_046	0	0	0	0	97	97	Heterocapsa circularisquama RNA virus, isolate: HcRNAV659, capsid protein	94.301	1.60E-76	LC120626.1
ASV_047	0	0	0	96	0	96	Nohit			
ASV_048	9	27	59	0	0	95	Nohit			
ASV_049	0	95	0	0	0	95	Nohit			
ASV_050	0	0	0	0	89	89	Heterocapsa circularisquama RNA virus, isolate: HcRNAV109	96.891	7.32E-85	AB218609.1
ASV_051	0	0	0	0	85	85	Heterocapsa circularisquama RNA virus, isolate: HcRNAV109	93.158	1.61E-71	AB218609.1
ASV_052	0	0	0	0	83	83	Nohit			
ASV_053	0	0	0	0	82	82	Nohit			
ASV_054	78	0	0	0	0	78	Nohit			
ASV_055	0	7	64	0	0	71	Nohit			
ASV_056	23	47	0	0	0	70	Nohit			
ASV_057	0	0	0	0	65	65	Nohit			
ASV_058	0	0	0	61	0	61	Heterocapsa circularisquama RNA virus, isolate: HcRNAV659, capsid protein	95.361	7.37E-80	LC120626.1
ASV_061	52	0	0	0	0	52	Nohit			
ASV_062	0	52	0	0	0	52	Nohit			
ASV_064	0	0	0	0	51	51	Heterocapsa circularisquama RNA virus, isolate: HcRNAV659, capsid protein	94.301	1.60E-76	LC120626.1
ASV_065	9	21	18	0	0	48	Uncultured bacterium AD243-H1	89.873	2.97E-48	EU686591.1
ASV_066	0	0	0	43	0	43	Heterocapsa circularisquama RNA virus, isolate: HcRNAV659, capsid protein	94.301	1.60E-76	LC120626.1
ASV_067	0	0	0	0	42	42	Heterocapsa circularisquama RNA virus, isolate: HcRNAV659, capsid protein	94.301	1.60E-76	LC120626.1
ASV_069	0	0	0	0	40	40	Heterocapsa circularisquama RNA virus, isolate: HcRNAV109	96.373	3.40E-83	AB218609.1
ASV_071	0	0	0	0	38	38	Beihai narna-like virus 9 strain HOU146315	85.915	3.20E-09	KX883534.1
ASV_072	0	38	0	0	0	38	Nohit			
ASV_074	0	37	0	0	0	37	Nohit			
ASV_076	0	2	0	32	0	34	Nohit			
ASV_078	9	5	15	4	0	33	Uncultured bacterium clone F5K2Q4C04JL08B, 23S ribosomal RNA gene	87.975	1.09E-42	GU926844.1
ASV_079	15	17	0	0	0	32	Nohit			
ASV_080	31	0	0	0	0	31	Nohit			
ASV_081	0	0	0	0	31	31	*Gemmata* sp. SH-PL17	80.569	5.26E-32	CP011271.1
ASV_082	0	0	0	30	0	30	Heterocapsa circularisquama RNA virus, isolate: HcRNAV659, capsid protein	94.301	1.60E-76	LC120626.1
ASV_083	0	0	0	0	12	12	Nohit			
ASV_084	0	0	0	29	0	29	Nohit			
ASV_085	0	0	0	28	0	28	Nohit			
ASV_088	0	0	22	0	0	22	Heterocapsa circularisquama RNA virus, isolate: HcRNAV109	92.632	7.47E-70	AB218609.1
ASV_089	0	0	0	22	0	22	Nohit			
ASV_092	0	0	6	15	0	21	Nohit			
ASV_095	0	0	0	0	19	19	Nohit			
ASV_096	0	5	14	0	0	19	Nohit			
ASV_099	18	0	0	0	0	18	Nohit			
ASV_100	0	0	0	17	0	17	Nohit			
ASV_102	16	0	0	0	0	16	Nohit			
ASV_103	0	0	16	0	0	16	Nohit			
ASV_105	0	15	0	0	0	15	Nohit			
ASV_106	0	0	15	0	0	15	Nohit			
ASV_109	0	12	0	0	0	12	Nohit			
ASV_110	0	0	0	0	11	11	Nohit			
ASV_116	0	0	0	0	9	9	*Methanotorris igneus* strain Kol 5, 23S ribosomal RNA gene	83.41	2.72E-46	NR_103020.1
ASV_117	0	0	0	0	8	8	Nohit			
ASV_118	0	0	0	8	0	8	Nohit			
ASV_121	0	0	7	0	0	7	Nohit			
ASV_124	0	0	0	0	6	6	Nohit			
ASV_125	0	0	0	0	6	6	Nohit			
ASV_126	0	0	6	0	0	6	Nohit			
ASV_127	0	0	6	0	0	6	Nohit			
ASV_128	0	0	6	0	0	6	Nohit			
ASV_129	0	0	0	6	0	6	Nohit			
ASV_130	5	0	0	0	0	5	Nohit			
ASV_131	0	0	5	0	0	5	Nohit			
ASV_132	0	0	5	0	0	5	Nohit			
ASV_133	0	0	5	0	0	5	Nohit			
ASV_134	0	0	0	5	0	5	Nohit			
ASV_139	0	4	0	0	0	4	Nohit			
ASV_140	0	0	4	0	0	4	Nohit			
ASV_141	0	0	4	0	0	4	Nohit			
ASV_142	0	0	4	0	0	4	Uncultured archaeon clone SCSjl2238, 16S ribosomal RNA gene	94.805	7.02E-132	KU351216.1
ASV_143	0	0	4	0	0	4	Nohit			
ASV_144	0	0	0	4	0	4	Nohit			
ASV_152	3	0	0	0	0	3	Jenufa perforata culture-collection CAUP:H 8101 chloroplast	95.455	9.93E-09	KT625413.1
ASV_153	3	0	0	0	0	3	Nohit			
ASV_154	0	0	0	0	3	3	*Frankia inefficax* strain EuI1c	80.153	2.30E-15	CP002299.1
ASV_155	0	3	0	0	0	3	*Geoalkalibacter subterraneus* strain Red1	85.664	2.54E-76	CP010311.1
ASV_156	0	3	0	0	0	3	Nohit			
ASV_157	0	0	3	0	0	3	*Streptomyces* sp. CdTB01	94.286	0.001	CP013743.1
ASV_158	0	0	3	0	0	3	Nohit			
ASV_159	0	0	3	0	0	3	Nohit			
ASV_160	0	0	3	0	0	3	Nohit			
ASV_167	2	0	0	0	0	2	Nohit			
ASV_168	0	2	0	0	0	2	Nohit			
ASV_169	0	2	0	0	0	2	*Lysobacter maris* strain HZ9B chromosome	81.724	1.26E-59	CP029843.1
ASV_170	0	2	0	0	0	2	Nohit			
ASV_171	0	0	2	0	0	2	Nohit			
ASV_172	0	0	2	0	0	2	Nohit			
ASV_173	0	0	2	0	0	2	Nohit			
ASV_174	0	0	2	0	0	2	Nohit			
ASV_175	0	0	2	0	0	2	Nohit			
ASV_176	0	0	2	0	0	2	Nohit			
ASV_177	0	0	0	2	0	2	*Methanotorris igneus* strain Kol 5, 23S ribosomal RNA gene	84.39	2.72E-46	NR_103020.1
ASV_178	0	0	0	2	0	2	Nohit			

Table S3 List of amplicon sequence variants (ASVs) coding the same amino acid sequence.

Merged ASV #	ASV #	Amino acid sequence
ASV_001s	001, 005, 007, 008, 039, 050, 069	LALRGVQGDSYPLNMSALSNFNCVHDVTADKLTWTDYAGFYPTGLAPIVFVNEAKQTMNYLVSV
ASV_002s	002, 004, 006, 010, 012, 013, 015, 017, 018, 019, 021, 022, 025, 027, 030, 033, 037, 041, 047, 049, 054, 057, 062	LALRGVQLDSYPLDMSALADFRAVLKAADAQFTWDSANRGCFSGWAPLVIINDAANDLELDFLITI
ASV_003s	003, 016, 024, 036, 038, 042, 058, 066, 082	LALRGVQGDSYPLNMSALSNFNCLTDVAEGQISWTDSTGFYPAGLAPIVFVNEAKQTMNYLVSV
ASV_009s	009, 035	LALRGVQGDSYPLNMSALSNFNCLTDVAEGKLSWTDSSGHYPAGLAPIVFVNEAKQTMNYLVSV
ASV_011s	011, 051	LALRGVQGDSYPLNMSALSNFNCLRDVAEGTLSWTDDVGFYPTGLAPIVFVNEAKQTMNYLVSI
ASV_023s	023, 105	LALRGVQADSYPLNMSALASFLPVNNDTSGTITYTNAGWPGAFRGFAPIVIVNTGSTLDPPLMLQFLVTV
ASV_032s	032, 117	LTLRGVQMDSHPLSMADISSFDPMLYDTPNPSFTGAGEIIGAWDVNGPYPCGWAPMAFVNPLGAEIQLLIGV
ASV_040s	040, 084, 102	LVLRGVQMDSHPLSMADVSDFRGMFELTSSLNQTGSAPGGWSASSGNIDPEGWAPMVIYNPNNTPLSLLVSI
ASV_048s	048, 118	LSLRGVQMDSHPLSMADVSEFLPMEPVAEPFTPSDGWKGSDSPQFCGWAPMAIYNPDKADLSILVSI
ASV_056s	056, 061, 072, 080, 131	LSLRGIQADSYPLNMNALSDFLEVRDLAPGVVTMDSHKFDGHPVGFAPIVIVNEGSTADPPLMLNFLVTI
ASV_076s	076, 159	LALKGVQIDCVPFDMNAMSDFKTRFPSTSGSYTNNDPAIRFDGLAPIFVYNPEEIDLQYLVCC
ASV_079s	079, 121	LALRGVQMDSYPLNMSALADFRPVLDSVDVTTTWSTANLTHPLGFAPIVVINEASADDPPLALNFLVSV
ASV_099s	099, 109, 128	LALRGVQMNSHPLSMADVSEFLPMLTKEPGTSNNEAWTDASPHPVGWAPIMVYNPDNAHLTFMVSI
ASV_100s	100, 106	LALRGVQLDSYPLDMNKLSDFRPILGVADTNFTWNENARIHPAGFAPIVIINQASGEVPPLSLNLLITV
ASV_127s	127, 139	LALRGVQIDSFPLNMAEVSNFTPLRKIGDRATATFSDNSMEPRGWAPIVIYNGGAGGADGLELELLITV

Table S4 Frequency and abundance of reads at each sample subdivision depth and BLASTp hit table. Colored rows are used for phylogenetic analysis as HcRNAV-related sequence.

ASV ID*	Read count in each depth of sediment					Subtotal reads	Abundance in total reads	Top hit of BLASTp search			
	0-3 cm	3-6 cm	6-9 cm	9-12 cm	12-15 cm			Target	Identity (%)	E-value	Accession number
ASV_002s	39434	41500	35680	12499	9455	138568	79.34	hypothetical protein [Beihai narna-like virus 9]	56.5	4.00E-14	YP_009333317.1
ASV_003s	887	483	626	13758	8399	24153	13.83	major viral capsid protein, partial [Heterocapsa circularisquama RNA virus 01]	100	2.00E-36	BCI98894.1
ASV_001s	253	176	179	4384	2361	7353	4.21	major viral capsid protein, partial [Heterocapsa circularisquama RNA virus 01]	100	2.00E-37	BCI98817.1
ASV_011s	48	21	0	964	221	1254	0.72	major viral capsid protein, partial [Heterocapsa circularisquama RNA virus 01]	89.1	4.00E-32	BCI98894.1
ASV_009s	32	20	38	276	170	536	0.31	major viral capsid protein, partial [Heterocapsa circularisquama RNA virus 01]	100	2.00E-36	BCI98853.1
ASV_020	0	0	0	373	0	373	0.21	major viral capsid protein, partial [Heterocapsa circularisquama RNA virus 01]	98.4	2.00E-35	BCI98876.1
ASV_023s	26	15	262	21	0	324	0.19	hypothetical protein [Beihai narna-like virus 9]	60	1.00E-13	YP_009333317.1
ASV_031	0	0	0	0	228	228	0.13	major viral capsid protein, partial [Heterocapsa circularisquama RNA virus 01]	98.4	9.00E-36	BCI98894.1
ASV_056s	106	85	5	0	0	196	0.11	hypothetical protein [Beihai narna-like virus 9]	55.7	8.00E-14	YP_009333317.1
ASV_034	0	0	0	0	174	174	0.1	major viral capsid protein, partial [Heterocapsa circularisquama RNA virus 01]	98.4	1.00E-35	BCI98894.1
ASV_040s	16	15	112	29	0	172	0.1	hypothetical protein 2 [Beihai sobemo-like virus 7]	65.3	1.00E-17	YP_009336926.1
ASV_048s	9	27	59	8	0	103	0.06	hypothetical protein [Beihai narna-like virus 9]	50.7	5.00E-13	YP_009333317.1
ASV_043	4	4	94	0	0	102	0.06	hypothetical protein [Beihai narna-like virus 9]	53.6	5.00E-11	YP_009333317.1
ASV_046	0	0	0	0	97	97	0.06	major viral capsid protein, partial [Heterocapsa circularisquama RNA virus 01]	98.4	2.00E-35	BCI98894.1
ASV_052	0	0	0	0	83	83	0.05	hypothetical protein [Beihai narna-like virus 9]	55.1	1.00E-13	YP_009333317.1
ASV_053	0	0	0	0	82	82	0.05	hypothetical protein [Beihai narna-like virus 9]	55.1	3.00E-13	YP_009333317.1
ASV_055	0	7	64	0	0	71	0.04	hypothetical protein 2 [Beihai sobemo-like virus 9]	47.2	4.00E-08	YP_009336950.1
ASV_064	0	0	0	0	51	51	0.03	major viral capsid protein, partial [Heterocapsa circularisquama RNA virus 01]	98.4	9.00E-36	BCI98894.1
ASV_065	9	21	18	0	0	48	0.03	Nohit			
ASV_067	0	0	0	0	42	42	0.02	major viral capsid protein, partial [Heterocapsa circularisquama RNA virus 01]	98.4	5.00E-35	BCI98894.1
ASV_079s	15	17	7	0	0	39	0.02	hypothetical protein [Beihai narna-like virus 9]	66.7	8.00E-23	YP_009333317.1
ASV_071	0	0	0	0	38	38	0.02	hypothetical protein [Beihai narna-like virus 9]	66.7	4.00E-23	YP_009333317.1
ASV_074	0	37	0	0	0	37	0.02	hypothetical protein [Beihai narna-like virus 9]	58	5.00E-15	YP_009333317.1
ASV_076s	0	2	3	32	0	37	0.02	hypothetical protein [Beihai narna-like virus 10]	49.2	6.00E-13	YP_009333272.1
ASV_099s	18	12	6	0	0	36	0.02	hypothetical protein [Beihai weivirus-like virus 21]	46.2	8.00E-10	APG78109.1
ASV_078	9	5	15	4	0	33	0.019	Nohit			
ASV_100s	0	0	15	17	0	32	0.018	hypothetical protein [Beihai narna-like virus 9]	60.9	3.00E-20	YP_009333317.1
ASV_081	0	0	0	0	31	31	0.018	Nohit			
ASV_085	0	0	0	28	0	28	0.016	hypothetical protein 2 [Beihai sobemo-like virus 9]	52.9	1.00E-09	YP_009336950.1
ASV_026	0	0	23	0	0	23	0.013	hypothetical protein 2 [Beihai sobemo-like virus 7]	61.1	7.00E-23	YP_009336926.1
ASV_088	0	0	22	0	0	22	0.013	major viral capsid protein, partial [Heterocapsa circularisquama RNA virus 01]	87.5	1.00E-31	BCI98894.1
ASV_089	0	0	0	22	0	22	0.013	major viral capsid protein, partial [Heterocapsa circularisquama RNA virus 01]	54.7	4.00E-17	BCI98905.1
ASV_092	0	0	6	15	0	21	0.012	major viral capsid protein, partial [Heterocapsa circularisquama RNA virus 01]	59.4	5.00E-19	BCI98889.1
ASV_032s	0	0	0	12	8	20	0.011	hypothetical protein 2 [Beihai sobemo-like virus 9]	47.2	4.00E-08	YP_009336950.1
ASV_095	0	0	0	0	19	19	0.011	Nohit			
ASV_096	0	5	14	0	0	19	0.011	putative capsid protein [Beihai sobemo-like virus 8]	71.6	7.00E-27	YP_009336986.1
ASV_103	0	0	16	0	0	16	0.009	hypothetical protein 2 [Beihai sobemo-like virus 5]	75	1.00E-24	YP_009336901.1
ASV_083	0	0	0	0	12	12	0.007	hypothetical protein 2 [Beihai sobemo-like virus 9]	52.1	2.00E-12	YP_009336950.1
ASV_110	0	0	0	0	11	11	0.006	hypothetical protein 2 [Beihai sobemo-like virus 9]	76.1	2.00E-28	YP_009336950.1
ASV_127s	0	4	6	0	0	10	0.006	hypothetical protein [Beihai weivirus-like virus 21]	65.4	6.00E-13	YP_009337073.1
ASV_116	0	0	0	0	9	9	0.005	Nohit			
ASV_045	0	7	0	0	0	7	0.004	hypothetical protein [Beihai weivirus-like virus 16]	47.7	4.00E-09	YP_009336993.1
ASV_124	0	0	0	0	6	6	0.003	Nohit			
ASV_125	0	0	0	0	6	6	0.003	Gfo/Idh/MocA family oxidoreductase [Desulfobacterales bacterium]	88.1	4.00E-59	NNL76790.1
ASV_126	0	0	6	0	0	6	0.003	putative capsid protein [Beihai weivirus-like virus 17]	52.3	1.00E-09	YP_009336945.1
ASV_129	0	0	0	6	0	6	0.003	putative capsid [Barns Ness breadcrumb sponge weivirus-like virus 1]	54.6	2.00E-05	ASM94030.1
ASV_130	5	0	0	0	0	5	0.003	hypothetical protein 2 [Beihai sobemo-like virus 4]	65.6	1.00E-19	YP_009336877.1
ASV_132	0	0	5	0	0	5	0.003	hypothetical protein [Beihai narna-like virus 9]	49.3	2.00E-08	YP_009333317.1
ASV_133	0	0	5	0	0	5	0.003	hypothetical protein [Beihai narna-like virus 9]	60	4.00E-14	YP_009333317.1
ASV_134	0	0	0	5	0	5	0.003	hypothetical protein [Beihai narna-like virus 9]	68.1	1.00E-22	YP_009333317.1
ASV_140	0	0	4	0	0	4	0.002	hypothetical protein [Beihai weivirus-like virus 21]	44.9	8.00E-09	YP_009337073.1
ASV_141	0	0	4	0	0	4	0.002	hypothetical protein 2 [Beihai sobemo-like virus 7]	61.1	7.00E-22	YP_009336926.1
ASV_142	0	0	4	0	0	4	0.002	Nohit			
ASV_143	0	0	4	0	0	4	0.002	hypothetical protein 2 [Beihai sobemo-like virus 9]	52.1	3.00E-12	YP_009336950.1
ASV_144	0	0	0	4	0	4	0.002	hypothetical protein 2 [Beihai sobemo-like virus 7]	73.6	4.00E-26	YP_009336926.1
ASV_152	3	0	0	0	0	3	0.002	Nohit			
ASV_153	3	0	0	0	0	3	0.002	hypothetical protein 2 [Beihai sobemo-like virus 7]	56	1.00E-15	YP_009336926.1
ASV_154	0	0	0	0	3	3	0.002	phosphotransferase [Gammaproteobacteria bacterium]	92.3	2.00E-38	NIP89023.1
ASV_155	0	3	0	0	0	3	0.002	Nohit			
ASV_156	0	3	0	0	0	3	0.002	hypothetical protein 2 [Beihai sobemo-like virus 9]	53.5	3.00E-11	YP_009336950.1
ASV_157	0	0	3	0	0	3	0.002	Nohit			
ASV_158	0	0	3	0	0	3	0.002	Nohit			
ASV_160	0	0	3	0	0	3	0.002	hypothetical protein [Beihai weivirus-like virus 16]	51.5	9.00E-11	YP_009336993.1
ASV_167	2	0	0	0	0	2	0.001	Nohit			
ASV_168	0	2	0	0	0	2	0.001	Nohit			
ASV_169	0	2	0	0	0	2	0.001	Nohit			
ASV_170	0	2	0	0	0	2	0.001	Nohit			
ASV_171	0	0	2	0	0	2	0.001	hypothetical protein 2 [Beihai sobemo-like virus 7]	53.5	8.00E-10	YP_009336926.1
ASV_172	0	0	2	0	0	2	0.001	integrase arm-type DNA-binding domain-containing protein [Ruegeria arenilitoris]	65.7	5.00E-33	WP_170343491.1
ASV_173	0	0	2	0	0	2	0.001	hypothetical protein 2 [Beihai sobemo-like virus 5]	75	2.00E-24	YP_009336901.1
ASV_174	0	0	2	0	0	2	0.001	hypothetical protein [Beihai weivirus-like virus 21]	42.9	4.00E-06	YP_009337073.1
ASV_175	0	0	2	0	0	2	0.001	hypothetical protein 2 [Beihai sobemo-like virus 4]	75	1.00E-15	YP_009336877.1
ASV_176	0	0	2	0	0	2	0.001	putative capsid [Barns Ness breadcrumb sponge weivirus-like virus 10]	52.6	5.00E-14	ASM94042.1
ASV_177	0	0	0	2	0	2	0.001	Nohit			
ASV_178	0	0	0	2	0	2	0.001	hypothetical protein 2 [Beihai sobemo-like virus 7]	75	5.00E-28	YP_009336926.1

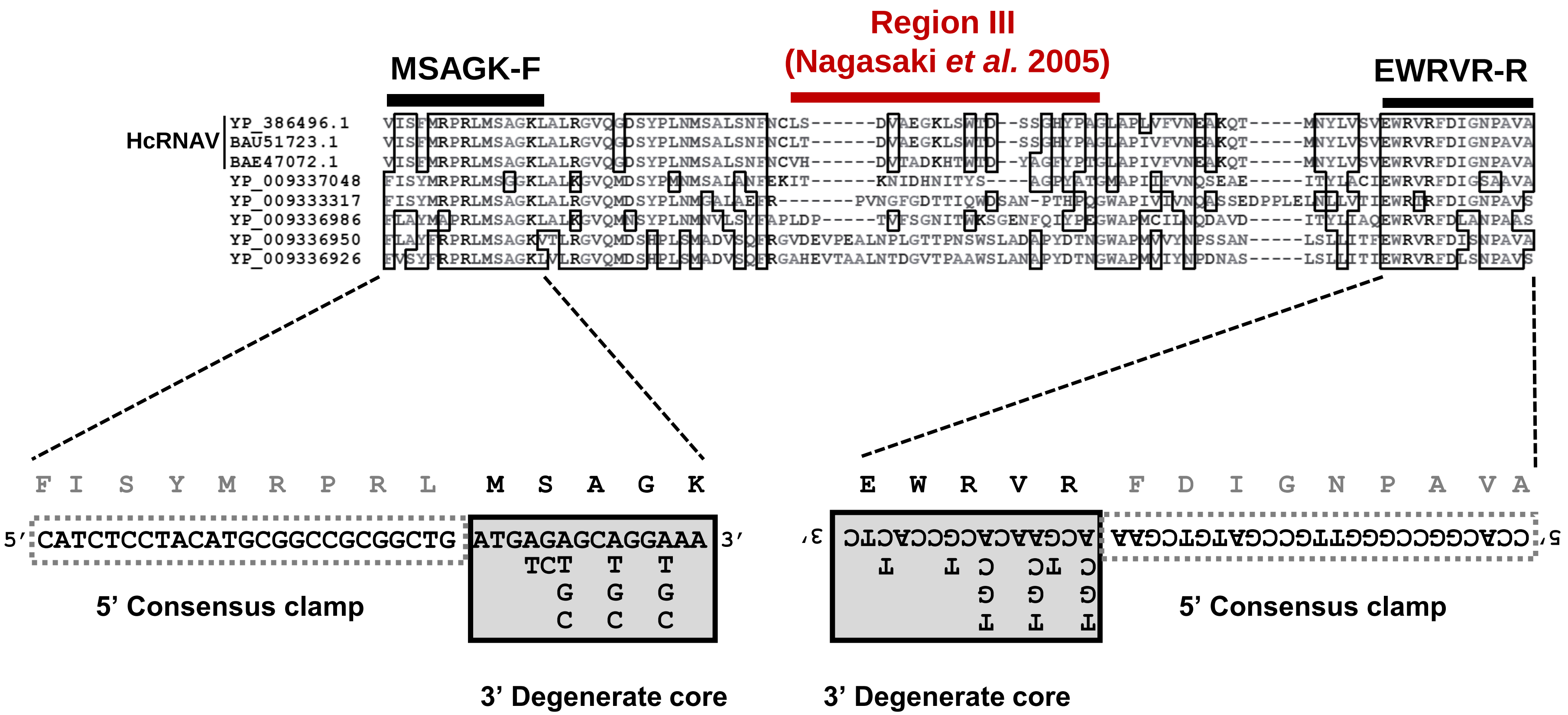

Fig. S1 Overview of the degenerate primer set designed in the present study. The MCP gene of HcRNAV (strains 34, 109, and 659) and hypothetical/putative capsid protein of viral sequences (Table S1) were used as reference sequences. The red colored region indicates one of the hypervariable regions in the MCP gene of HcRNAV identified by Nagasaki et al. (2005).
